# The “Toe Walking Tool”: a novel method of assessing toe walking children

**DOI:** 10.1186/1757-1146-4-S1-O51

**Published:** 2011-05-20

**Authors:** Cylie Williams, Paul Tinley, Michael Curtin

**Affiliations:** 1 Charles Sturt University, Albury, NSW, 2640, Australia; 2 Southern Health - Cardinia Casey Community Health, Cranbourne, VIC, 3977, Australia; 3 Peninsula Health – Community Health, Frankston, 3199, Australia

## Background

Idiopathic toe walking (ITW) can present in children above the age of three, and occurs in the absence of any medically diagnosed conditions. Identification of medical conditions that are associated with toe walking can be complex and a potentially daunting task for those that infrequently assess children. In order to facilitate entry for appropriate toe walking children into an ITW study, a tool was developed and validated.

## Methods

A review of literature established key indicators that may be found within the medical history and clinical presentation that identify an underlying condition potentially being responsible for the toe-walking gait. These key indicators were translated into a simple web based program or a paper version of 21 questions. The tool content was validated via a Delphi panel with medical and allied health professionals. It was then tested for reliability with twelve children and 10 allied health professionals.

## Results

The content of this tool and its design was determined valid by a Delphi panel process. Minor changes to order and wording were applied. The results of the reliability testing determine the tool to be reliable with a Fleiss Kappa=0.9028; z=29.6091; p<0.0001. This was calculated from 120 individual uses of the tool.

## Conclusions

There is a need for appropriate assessment tools for both training new clinicians working with the paediatric population, and for clinicians that infrequently assess children. The Toe Walking Tool is a valid and comprehensive way to assess children who toe walk. This tool will assist in the appropriate referral of children who may have a medical reason for their gait pattern and the identification of children with an ITW gait.

